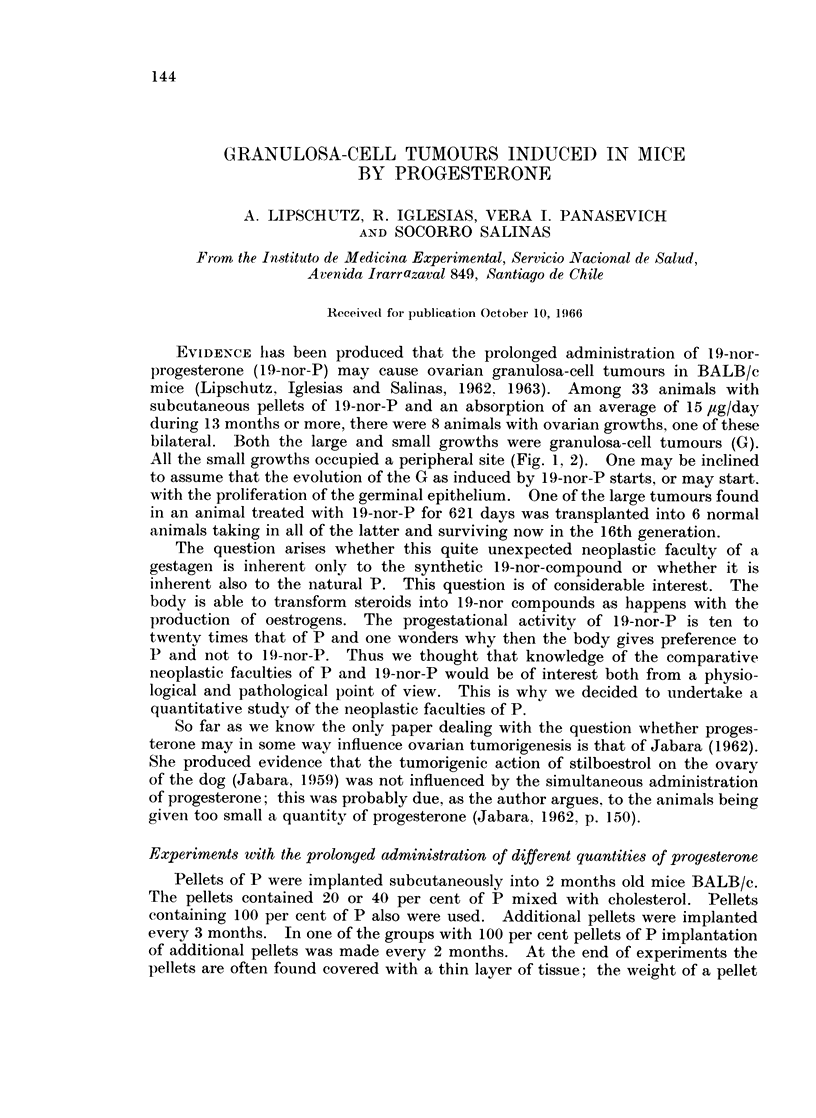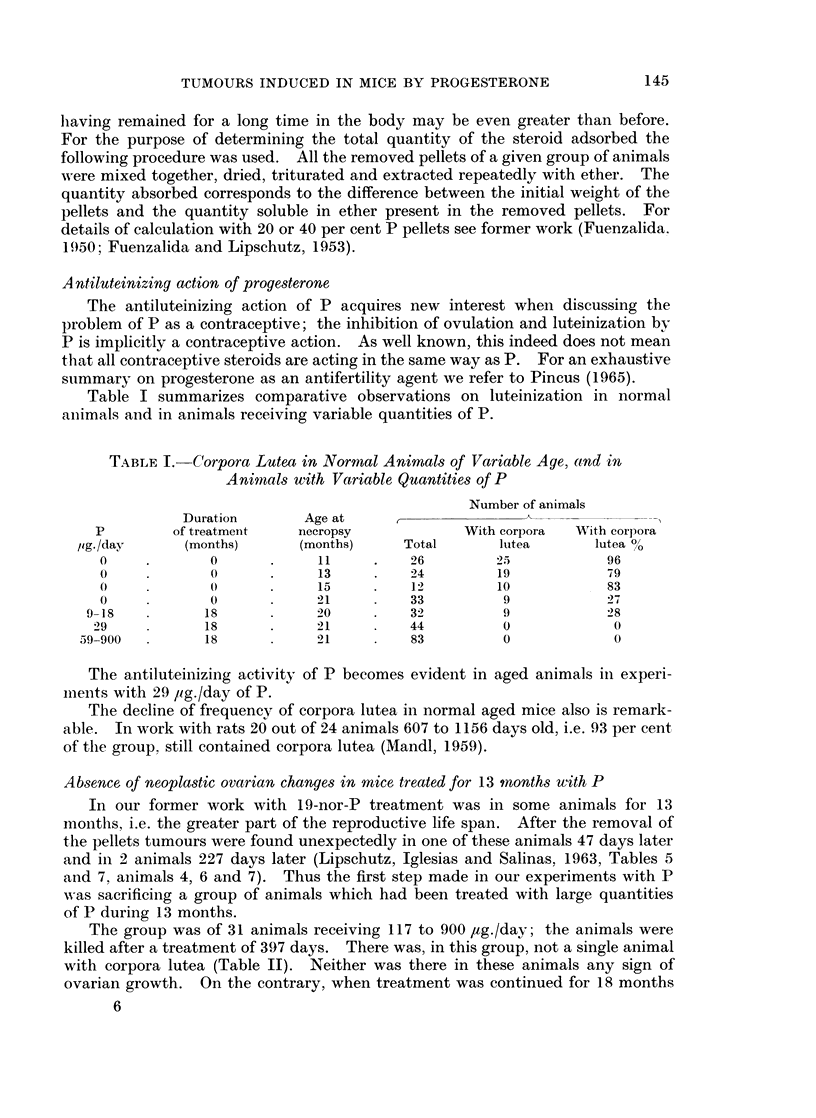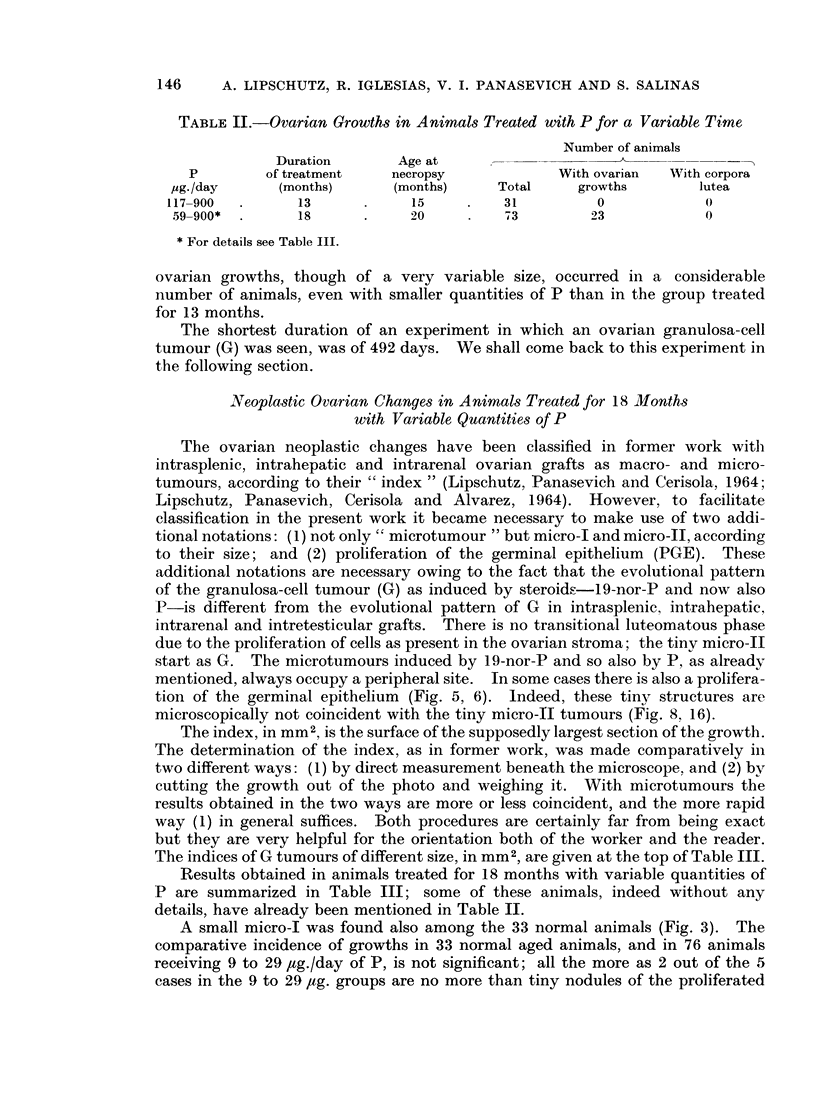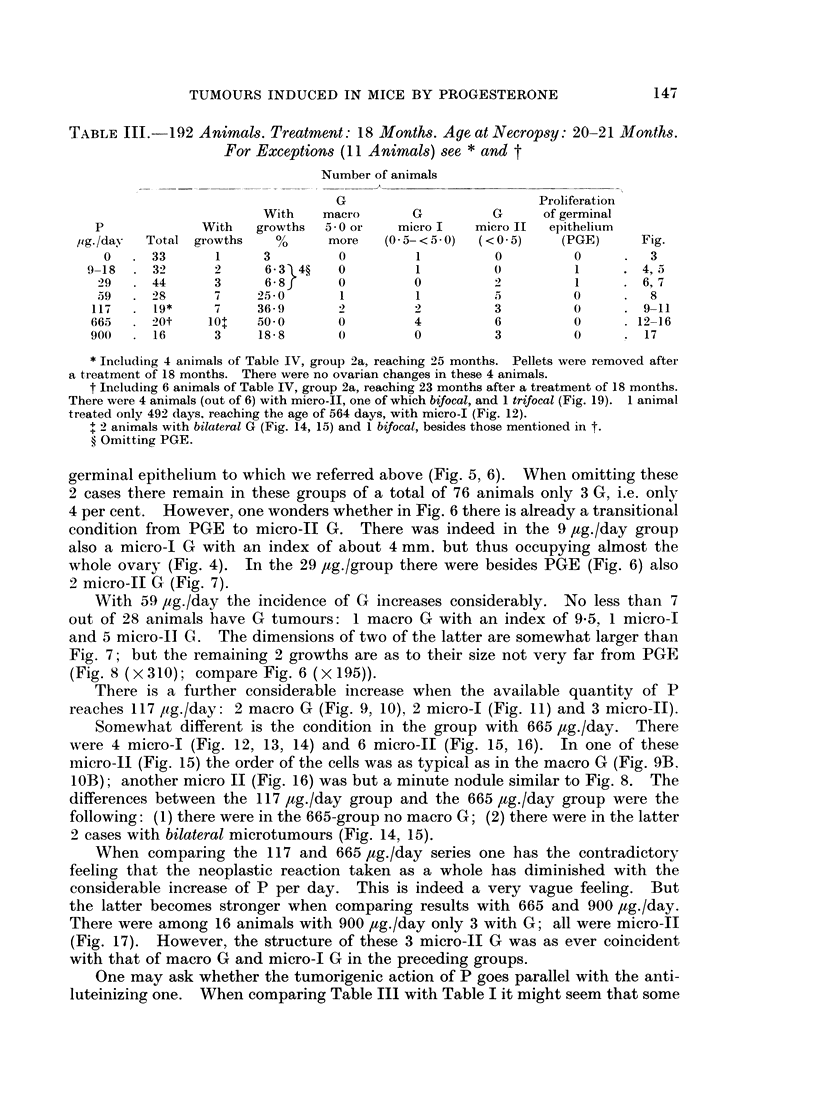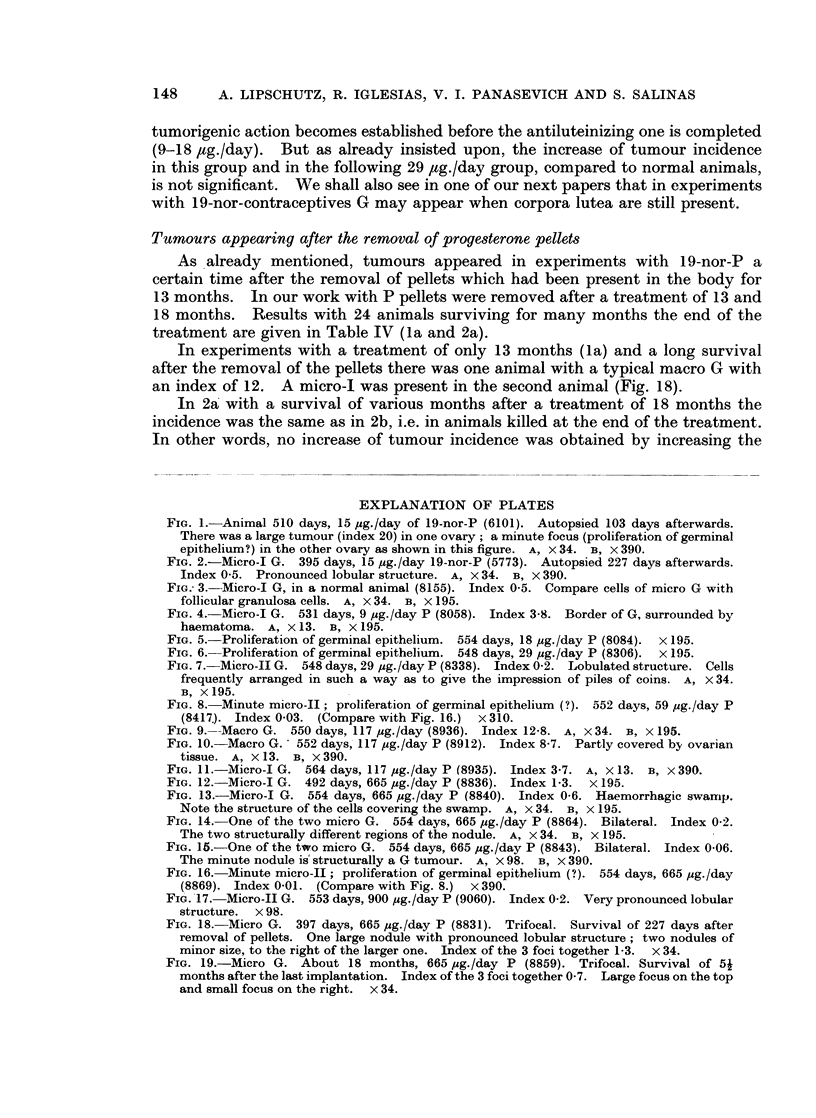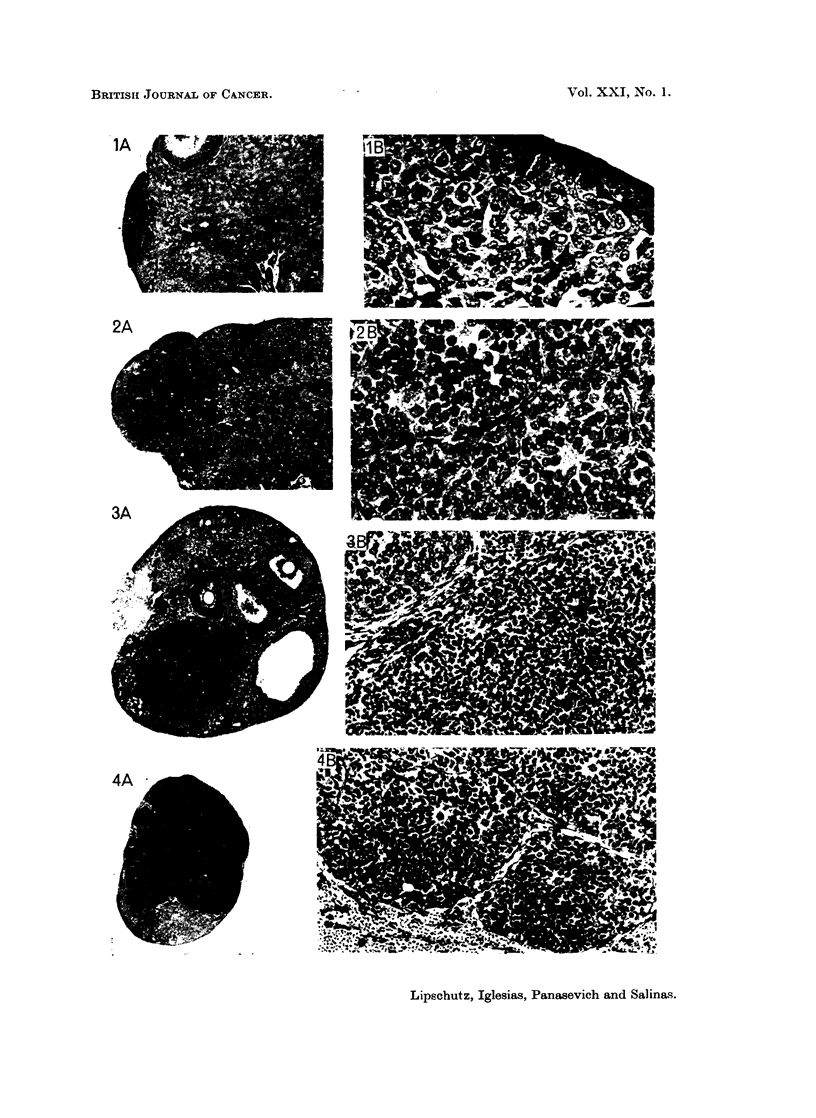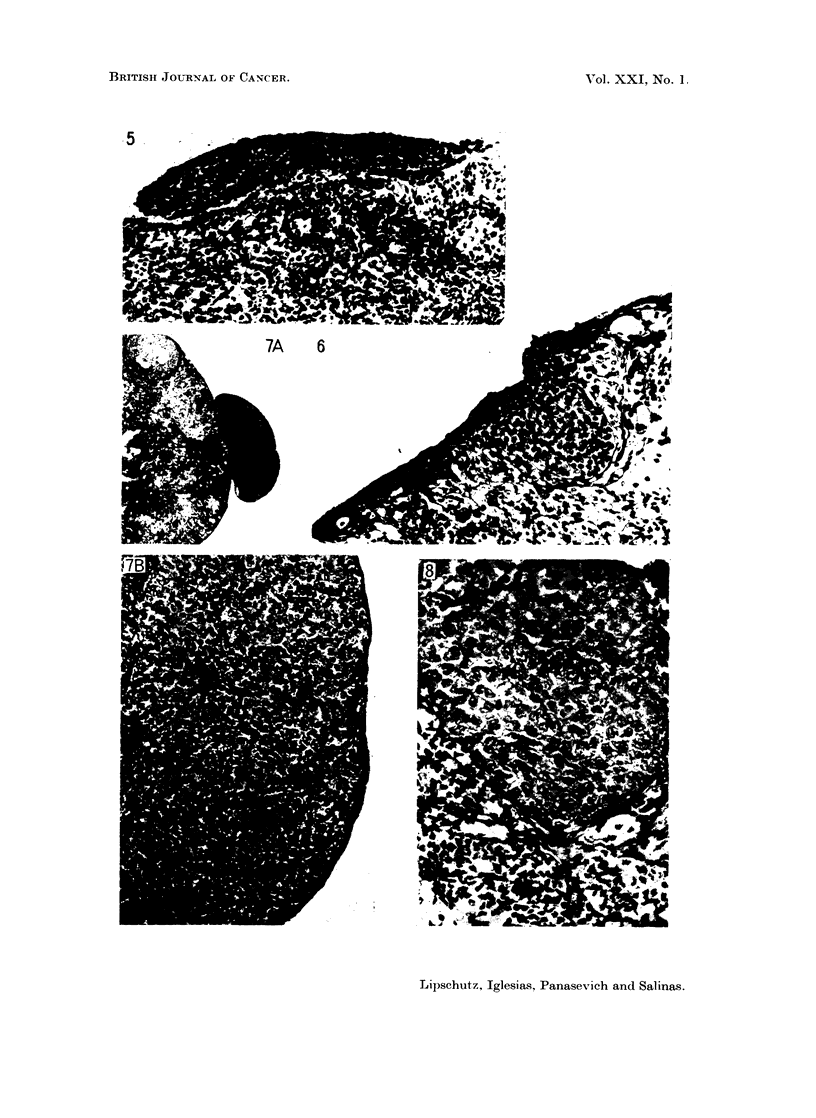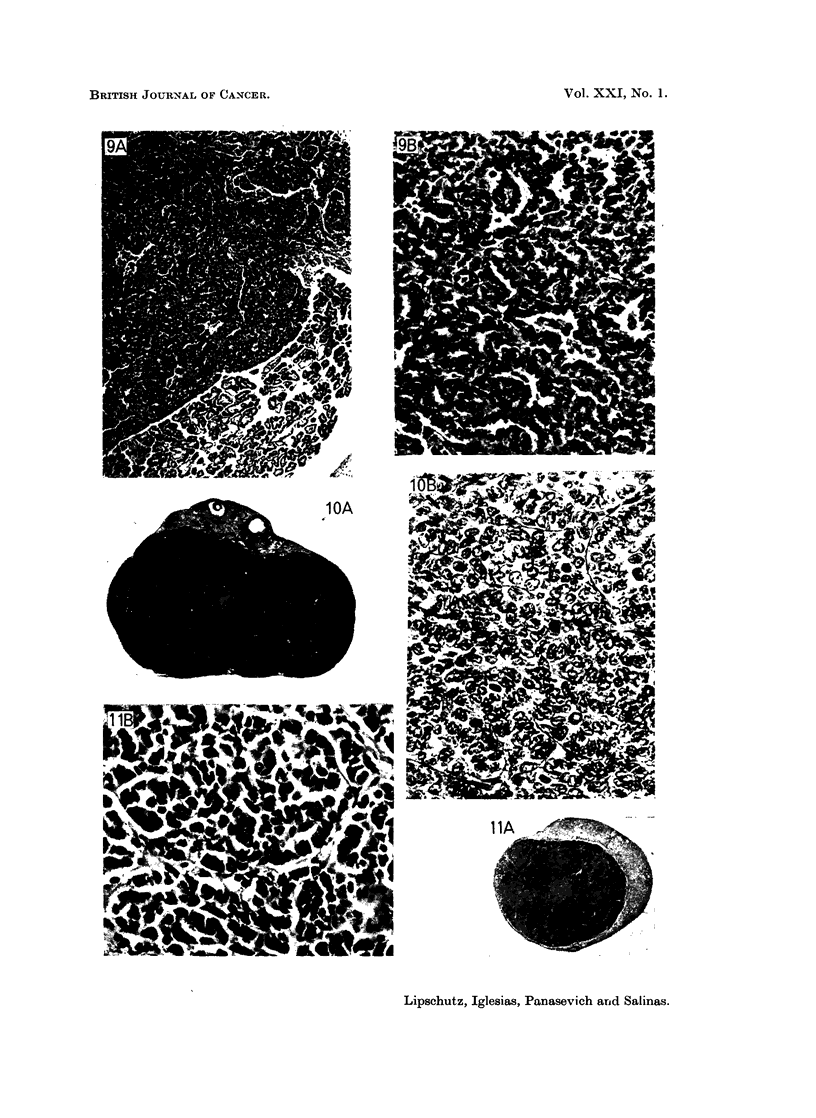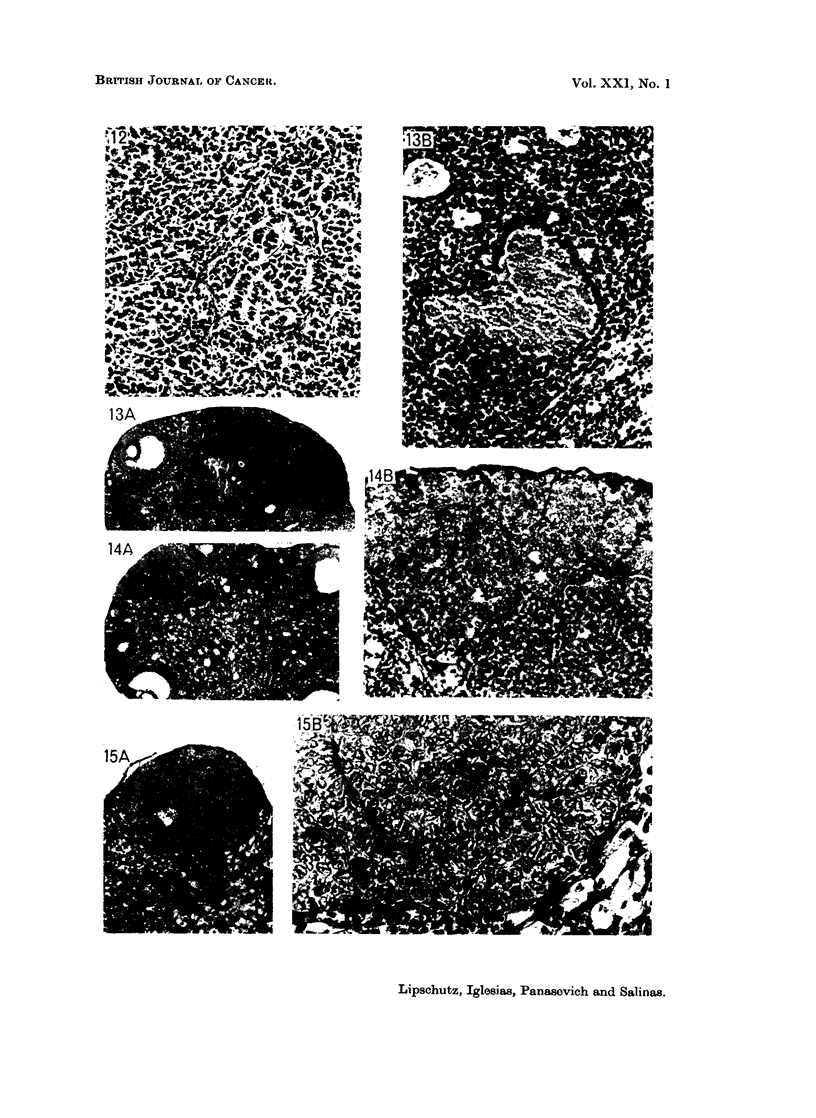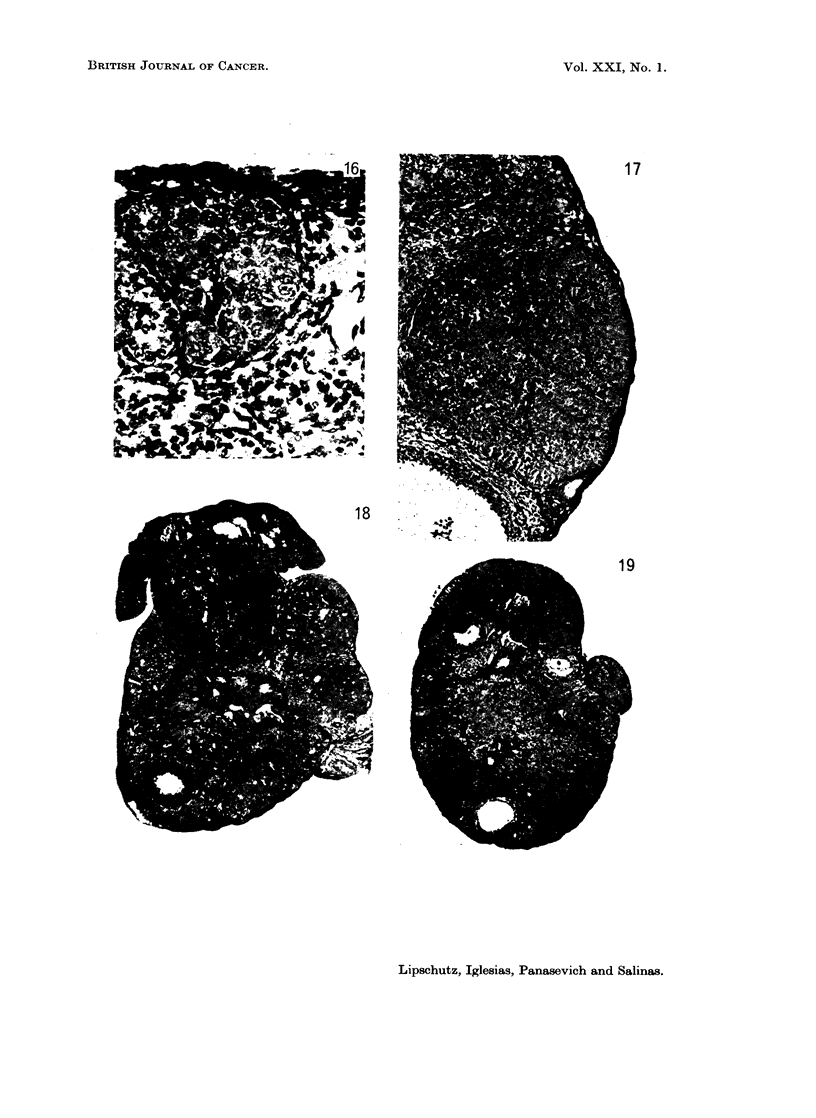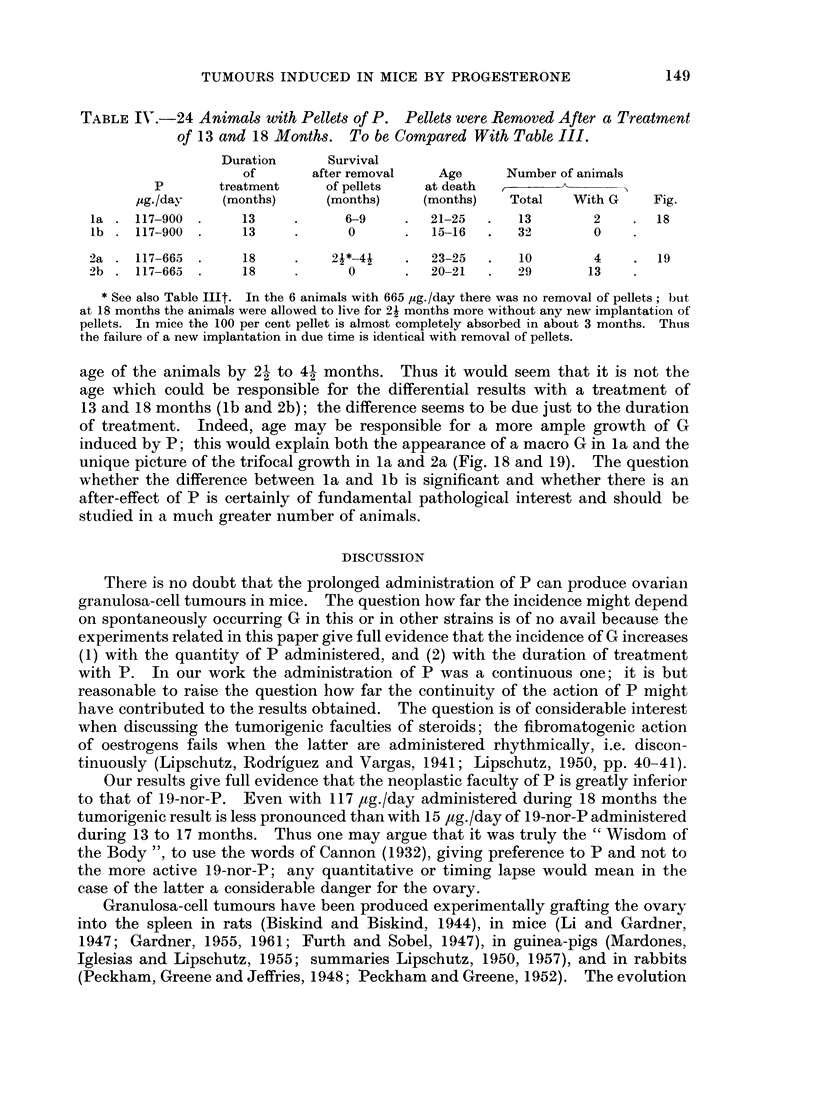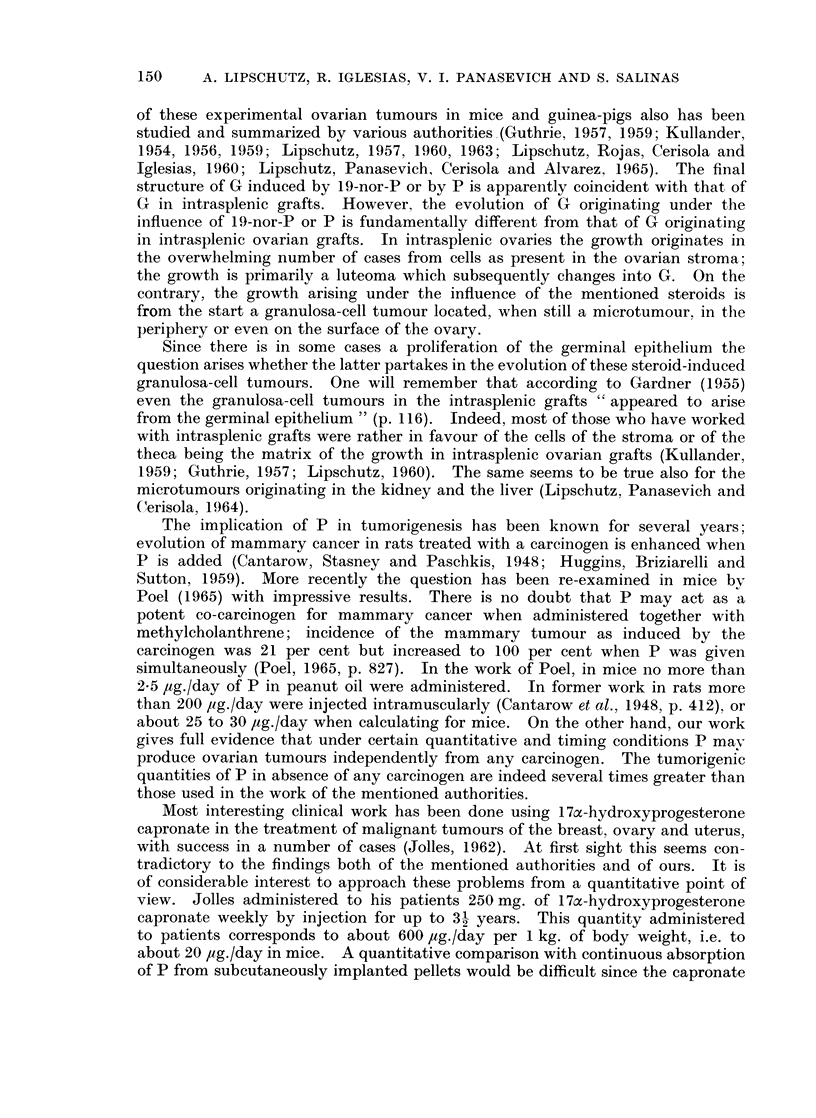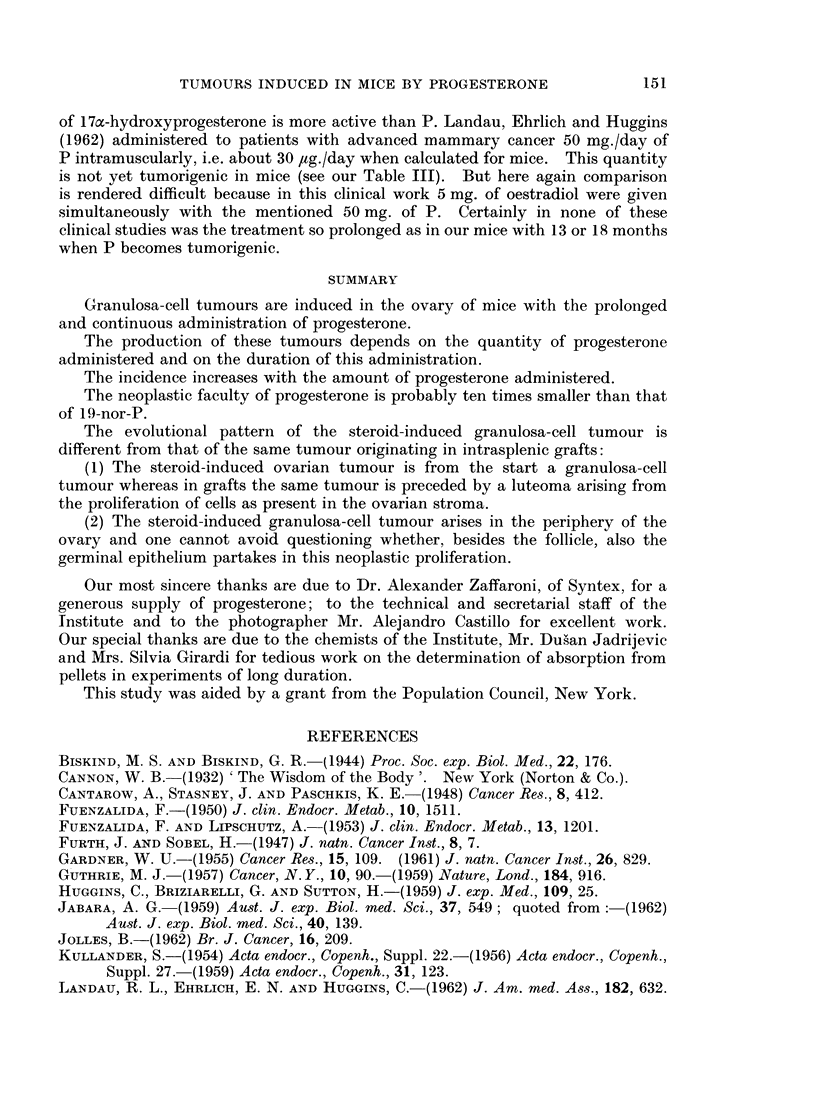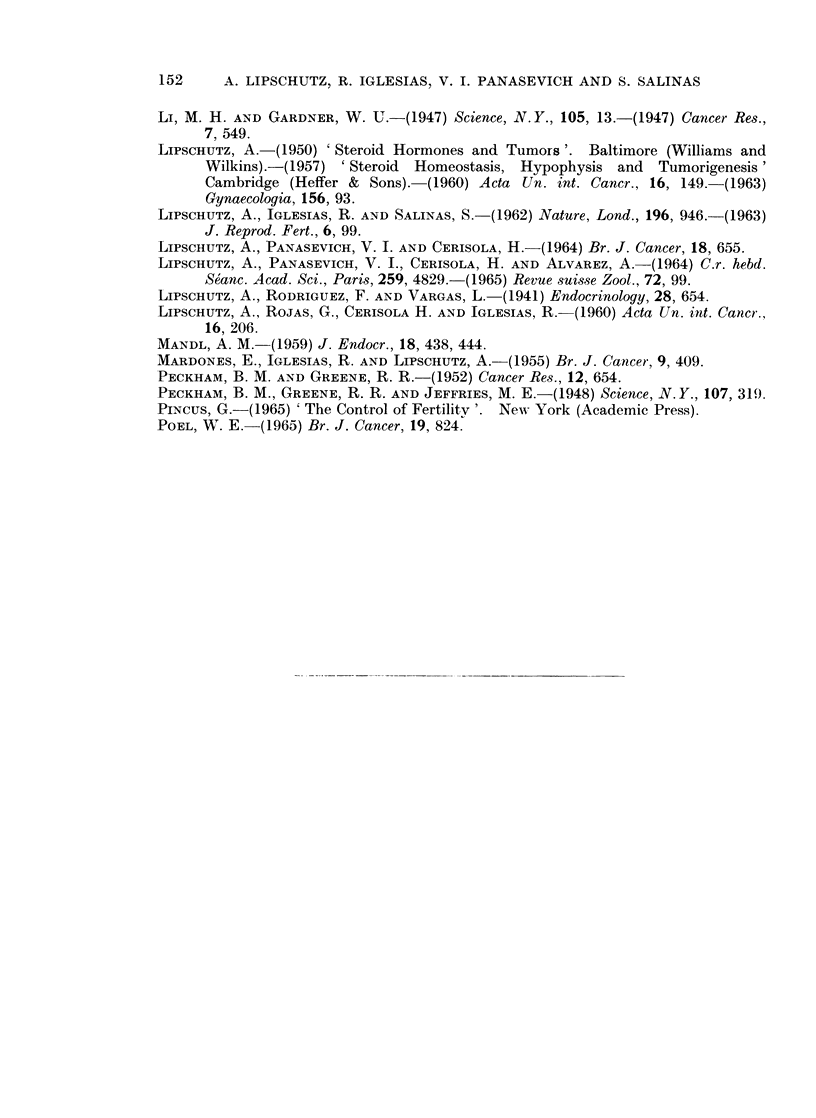# Granulosa-cell tumours induced in mice by progesterone.

**DOI:** 10.1038/bjc.1967.13

**Published:** 1967-03

**Authors:** A. Lipschutz, R. Iglesias, V. I. Panasevich, S. Salinas

## Abstract

**Images:**


### GRANULOSA-CELL TUMOURS INDUCED IN MICE BY PROGESTERONE

A. LIPSCHUTZ, R. IGLESIAS, VERA I. PANASEVICH AND SOCORRO SALINAS

*From the Instituto de Medicina Experimental, Servicio Nacional de Salud, Avenida Irarrazaval 849, Santiago de Chile*

Received for publication October 10, 1966

EVIDENCE has been produced that the prolonged administration of 19-nor-progesterone (19-nor-P) may cause ovarian granulosa-cell tumours in BALB/c mice (Lipschutz, Iglesias and Salinas, 1962, 1963). Among 33 animals with subcutaneous pellets of 19-nor-P and an absorption of an average of 15 $\mu$g/day during 13 months or more, there were 8 animals with ovarian growths, one of these bilateral. Both the large and small growths were granulosa-cell tumours (G). All the small growths occupied a peripheral site (Fig. 1, 2). One may be inclined to assume that the evolution of the G as induced by 19-nor-P starts, or may start, with the proliferation of the germinal epithelium. One of the large tumours found in an animal treated with 19-nor-P for 621 days was transplanted into 6 normal animals taking in all of the latter and surviving now in the 16th generation.

The question arises whether this quite unexpected neoplastic faculty of a gestagen is inherent only to the synthetic 19-nor-compound or whether it is inherent also to the natural P. This question is of considerable interest. The body is able to transform steroids into 19-nor compounds as happens with the production of oestrogens. The progestational activity of 19-nor-P is ten to twenty times that of P and one wonders why then the body gives preference to P and not to 19-nor-P. Thus we thought that knowledge of the comparative neoplastic faculties of P and 19-nor-P would be of interest both from a physiological and pathological point of view. This is why we decided to undertake a quantitative study of the neoplastic faculties of P.

So far as we know the only paper dealing with the question whether progesterone may in some way influence ovarian tumorigenesis is that of Jabara (1962). She produced evidence that the tumorigenic action of stilboestrol on the ovary of the dog (Jabara, 1959) was not influenced by the simultaneous administration of progesterone; this was probably due, as the author argues, to the animals being given too small a quantity of progesterone (Jabara, 1962, p. 150).

### *Experiments with the prolonged administration of different quantities of progesterone*

Pellets of P were implanted subcutaneously into 2 months old mice BALB/c. The pellets contained 20 or 40 per cent of P mixed with cholesterol. Pellets containing 100 per cent of P also were used. Additional pellets were implanted every 3 months. In one of the groups with 100 per cent pellets of P implantation of additional pellets was made every 2 months. At the end of experiments the pellets are often found covered with a thin layer of tissue; the weight of a pellet having remained for a long time in the body may be even greater than before. For the purpose of determining the total quantity of the steroid adsorbed the following procedure was used. All the removed pellets of a given group of animals were mixed together, dried, triturated and extracted repeatedly with ether. The quantity absorbed corresponds to the difference between the initial weight of the pellets and the quantity soluble in ether present in the removed pellets. For details of calculation with 20 or 40 per cent P pellets see former work (Fuenzalida. 1950; Fuenzalida and Lipschutz, 1953).

*Antiluteinizing action of progesterone*

The antiluteinizing action of P acquires new interest when discussing the problem of P as a contraceptive; the inhibition of ovulation and luteinization by P is implicitly a contraceptive action. As well known, this indeed does not mean that all contraceptive steroids are acting in the same way as P. For an exhaustive summary on progesterone as an antifertility agent we refer to Pincus (1965).

Table I summarizes comparative observations on luteinization in normal animals and in animals receiving variable quantities of P.

TABLE I.—*Corpora Lutea in Normal Animals of Variable Age, and in Animals with Variable Quantities of P*

| P μg./day | Duration of treatment (months) | Age at necropsy (months) | Number of animals: Total | With corpora lutea | With corpora lutea % |
|---|---|---|---|---|---|
| 0 | 0 | 11 | 26 | 25 | 96 |
| 0 | 0 | 13 | 24 | 19 | 79 |
| 0 | 0 | 15 | 12 | 10 | 83 |
| 0 | 0 | 21 | 33 | 9 | 27 |
| 9–18 | 18 | 20 | 32 | 9 | 28 |
| 29 | 18 | 21 | 44 | 0 | 0 |
| 59–900 | 18 | 21 | 83 | 0 | 0 |

The antiluteinizing activity of P becomes evident in aged animals in experiments with 29 μg./day of P.

The decline of frequency of corpora lutea in normal aged mice also is remarkable. In work with rats 20 out of 24 animals 607 to 1156 days old, i.e. 93 per cent of the group, still contained corpora lutea (Mandl, 1959).

*Absence of neoplastic ovarian changes in mice treated for* 13 *months with P*

In our former work with 19-nor-P treatment was in some animals for 13 months, i.e. the greater part of the reproductive life span. After the removal of the pellets tumours were found unexpectedly in one of these animals 47 days later and in 2 animals 227 days later (Lipschutz, Iglesias and Salinas, 1963, Tables 5 and 7, animals 4, 6 and 7). Thus the first step made in our experiments with P was sacrificing a group of animals which had been treated with large quantities of P during 13 months.

The group was of 31 animals receiving 117 to 900 μg./day; the animals were killed after a treatment of 397 days. There was, in this group, not a single animal with corpora lutea (Table II). Neither was there in these animals any sign of ovarian growth. On the contrary, when treatment was continued for 18 months

TABLE II.—*Ovarian Growths in Animals Treated with P for a Variable Time*

| P µg./day | Duration of treatment (months) | Age at necropsy (months) | Number of animals: Total | With ovarian growths | With corpora lutea |
|---|---|---|---|---|---|
| 117–900 | 13 | 15 | 31 | 0 | 0 |
| 59–900* | 18 | 20 | 73 | 23 | 0 |

* For details see Table III.

ovarian growths, though of a very variable size, occurred in a considerable number of animals, even with smaller quantities of P than in the group treated for 13 months.

The shortest duration of an experiment in which an ovarian granulosa-cell tumour (G) was seen, was of 492 days. We shall come back to this experiment in the following section.

### *Neoplastic Ovarian Changes in Animals Treated for* 18 *Months with Variable Quantities of P*

The ovarian neoplastic changes have been classified in former work with intrasplenic, intrahepatic and intrarenal ovarian grafts as macro- and micro-tumours, according to their " index " (Lipschutz, Panasevich and Cerisola, 1964; Lipschutz, Panasevich, Cerisola and Alvarez, 1964). However, to facilitate classification in the present work it became necessary to make use of two additional notations: (1) not only " microtumour " but micro-I and micro-II, according to their size; and (2) proliferation of the germinal epithelium (PGE). These additional notations are necessary owing to the fact that the evolutional pattern of the granulosa-cell tumour (G) as induced by steroids—19-nor-P and now also P—is different from the evolutional pattern of G in intrasplenic, intrahepatic, intrarenal and intretesticular grafts. There is no transitional luteomatous phase due to the proliferation of cells as present in the ovarian stroma; the tiny micro-II start as G. The microtumours induced by 19-nor-P and so also by P, as already mentioned, always occupy a peripheral site. In some cases there is also a proliferation of the germinal epithelium (Fig. 5, 6). Indeed, these tiny structures are microscopically not coincident with the tiny micro-II tumours (Fig. 8, 16).

The index, in $mm^2$, is the surface of the supposedly largest section of the growth. The determination of the index, as in former work, was made comparatively in two different ways: (1) by direct measurement beneath the microscope, and (2) by cutting the growth out of the photo and weighing it. With microtumours the results obtained in the two ways are more or less coincident, and the more rapid way (1) in general suffices. Both procedures are certainly far from being exact but they are very helpful for the orientation both of the worker and the reader. The indices of G tumours of different size, in $mm^2$, are given at the top of Table III.

Results obtained in animals treated for 18 months with variable quantities of P are summarized in Table III; some of these animals, indeed without any details, have already been mentioned in Table II.

A small micro-I was found also among the 33 normal animals (Fig. 3). The comparative incidence of growths in 33 normal aged animals, and in 76 animals receiving 9 to 29 µg./day of P, is not significant; all the more as 2 out of the 5 cases in the 9 to 29 µg. groups are no more than tiny nodules of the proliferated

TABLE III.—192 *Animals. Treatment:* 18 *Months. Age at Necropsy:* 20–21 *Months. For Exceptions* (11 *Animals*) *see* * *and* †

| | Number of animals | | | | | | | |
|---|---|---|---|---|---|---|---|---|
| P µg./day | Total | With growths | With growths % | G macro 5·0 or more | G micro I (0·5–<5·0) | G micro II (<0·5) | Proliferation of germinal epithelium (PGE) | Fig. |
| 0 | 33 | 1 | 3 | 0 | 1 | 0 | 0 | 3 |
| 9–18 | 32 | 2 | 6·3 } 4§ | 0 | 1 | 0 | 1 | 4, 5 |
| 29 | 44 | 3 | 6·8 } | 0 | 0 | 2 | 1 | 6, 7 |
| 59 | 28 | 7 | 25·0 | 1 | 1 | 5 | 0 | 8 |
| 117 | 19* | 7 | 36·9 | 2 | 2 | 3 | 0 | 9–11 |
| 665 | 20† | 10‡ | 50·0 | 0 | 4 | 6 | 0 | 12–16 |
| 900 | 16 | 3 | 18·8 | 0 | 0 | 3 | 0 | 17 |

* Including 4 animals of Table IV, group 2a, reaching 25 months. Pellets were removed after a treatment of 18 months. There were no ovarian changes in these 4 animals.

† Including 6 animals of Table IV, group 2a, reaching 23 months after a treatment of 18 months. There were 4 animals (out of 6) with micro-II, one of which *bifocal*, and 1 *trifocal* (Fig. 19). 1 animal treated only 492 days, reaching the age of 564 days, with micro-I (Fig. 12).

‡ 2 animals with *bilateral* G (Fig. 14, 15) and 1 *bifocal*, besides those mentioned in †.

§ Omitting PGE.

germinal epithelium to which we referred above (Fig. 5, 6). When omitting these 2 cases there remain in these groups of a total of 76 animals only 3 G, i.e. only 4 per cent. However, one wonders whether in Fig. 6 there is already a transitional condition from PGE to micro-II G. There was indeed in the 9 µg./day group also a micro-I G with an index of about 4 mm. but thus occupying almost the whole ovary (Fig. 4). In the 29 µg./group there were besides PGE (Fig. 6) also 2 micro-II G (Fig. 7).

With 59 µg./day the incidence of G increases considerably. No less than 7 out of 28 animals have G tumours: 1 macro G with an index of 9·5, 1 micro-I and 5 micro-II G. The dimensions of two of the latter are somewhat larger than Fig. 7; but the remaining 2 growths are as to their size not very far from PGE (Fig. 8 (×310); compare Fig. 6 (×195)).

There is a further considerable increase when the available quantity of P reaches 117 µg./day: 2 macro G (Fig. 9, 10), 2 micro-I (Fig. 11) and 3 micro-II).

Somewhat different is the condition in the group with 665 µg./day. There were 4 micro-I (Fig. 12, 13, 14) and 6 micro-II (Fig. 15, 16). In one of these micro-II (Fig. 15) the order of the cells was as typical as in the macro G (Fig. 9B, 10B); another micro II (Fig. 16) was but a minute nodule similar to Fig. 8. The differences between the 117 µg./day group and the 665 µg./day group were the following: (1) there were in the 665-group no macro G; (2) there were in the latter 2 cases with *bilateral* microtumours (Fig. 14, 15).

When comparing the 117 and 665 µg./day series one has the contradictory feeling that the neoplastic reaction taken as a whole has diminished with the considerable increase of P per day. This is indeed a very vague feeling. But the latter becomes stronger when comparing results with 665 and 900 µg./day. There were among 16 animals with 900 µg./day only 3 with G; all were micro-II (Fig. 17). However, the structure of these 3 micro-II G was as ever coincident with that of macro G and micro-I G in the preceding groups.

One may ask whether the tumorigenic action of P goes parallel with the anti-luteinizing one. When comparing Table III with Table I it might seem that some tumorigenic action becomes established before the antiluteinizing one is completed (9–18 μg./day). But as already insisted upon, the increase of tumour incidence in this group and in the following 29 μg./day group, compared to normal animals, is not significant. We shall also see in one of our next papers that in experiments with 19-nor-contraceptives G may appear when corpora lutea are still present.

*Tumours appearing after the removal of progesterone pellets*

As already mentioned, tumours appeared in experiments with 19-nor-P a certain time after the removal of pellets which had been present in the body for 13 months. In our work with P pellets were removed after a treatment of 13 and 18 months. Results with 24 animals surviving for many months the end of the treatment are given in Table IV (1a and 2a).

In experiments with a treatment of only 13 months (1a) and a long survival after the removal of the pellets there was one animal with a typical macro G with an index of 12. A micro-I was present in the second animal (Fig. 18).

In 2a with a survival of various months after a treatment of 18 months the incidence was the same as in 2b, i.e. in animals killed at the end of the treatment. In other words, no increase of tumour incidence was obtained by increasing the

---

EXPLANATION OF PLATES

FIG. 1.—Animal 510 days, 15 μg./day of 19-nor-P (6101). Autopsied 103 days afterwards. There was a large tumour (index 20) in one ovary ; a minute focus (proliferation of germinal epithelium?) in the other ovary as shown in this figure. A, ×34. B, ×390.

FIG. 2.—Micro-I G. 395 days, 15 μg./day 19-nor-P (5773). Autopsied 227 days afterwards. Index 0·5. Pronounced lobular structure. A, ×34. B, ×390.

FIG. 3.—Micro-I G, in a normal animal (8155). Index 0·5. Compare cells of micro G with follicular granulosa cells. A, ×34. B, ×195.

FIG. 4.—Micro-I G. 531 days, 9 μg./day P (8058). Index 3·8. Border of G, surrounded by haematoma. A, ×13. B, ×195.

FIG. 5.—Proliferation of germinal epithelium. 554 days, 18 μg./day P (8084). ×195.

FIG. 6.—Proliferation of germinal epithelium. 548 days, 29 μg./day P (8306). ×195.

FIG. 7.—Micro-II G. 548 days, 29 μg./day P (8338). Index 0·2. Lobulated structure. Cells frequently arranged in such a way as to give the impression of piles of coins. A, ×34. B, ×195.

FIG. 8.—Minute micro-II ; proliferation of germinal epithelium (?). 552 days, 59 μg./day P (8417). Index 0·03. (Compare with Fig. 16.) ×310.

FIG. 9.—Macro G. 550 days, 117 μg./day (8936). Index 12·8. A, ×34. B, ×195.

FIG. 10.—Macro G. 552 days, 117 μg./day P (8912). Index 8·7. Partly covered by ovarian tissue. A, ×13. B, ×390.

FIG. 11.—Micro-I G. 564 days, 117 μg./day P (8935). Index 3·7. A, ×13. B, ×390.

FIG. 12.—Micro-I G. 492 days, 665 μg./day P (8836). Index 1·3. ×195.

FIG. 13.—Micro-I G. 554 days, 665 μg./day P (8840). Index 0·6. Haemorrhagic swamp. Note the structure of the cells covering the swamp. A, ×34. B, ×195.

FIG. 14.—One of the two micro G. 554 days, 665 μg./day P (8864). Bilateral. Index 0·2. The two structurally different regions of the nodule. A, ×34. B, ×195.

FIG. 15.—One of the two micro G. 554 days, 665 μg./day P (8843). Bilateral. Index 0·06. The minute nodule is structurally a G tumour. A, ×98. B, ×390.

FIG. 16.—Minute micro-II ; proliferation of germinal epithelium (?). 554 days, 665 μg./day (8869). Index 0·01. (Compare with Fig. 8.) ×390.

FIG. 17.—Micro-II G. 553 days, 900 μg./day P (9060). Index 0·2. Very pronounced lobular structure. ×98.

FIG. 18.—Micro G. 397 days, 665 μg./day P (8831). Trifocal. Survival of 227 days after removal of pellets. One large nodule with pronounced lobular structure ; two nodules of minor size, to the right of the larger one. Index of the 3 foci together 1·3. ×34.

FIG. 19.—Micro G. About 18 months, 665 μg./day P (8859). Trifocal. Survival of 5½ months after the last implantation. Index of the 3 foci together 0·7. Large focus on the top and small focus on the right. ×34.

Lipschutz, Iglesias, Panasevich and Salinas.

Lipschutz, Iglesias, Panasevich and Salinas.

9A
9B
10A
10B
11B
11A

Lipschutz, Iglesias, Panasevich and Salinas.

Lipschutz, Iglesias, Panasevich and Salinas.

TABLE IV.—24 *Animals with Pellets of P. Pellets were Removed After a Treatment of* 13 *and* 18 *Months. To be Compared With Table III.*

| | P µg./day | Duration of treatment (months) | Survival after removal of pellets (months) | Age at death (months) | Number of animals: Total | Number of animals: With G | Fig. |
|---|---|---|---|---|---|---|---|
| 1a | 117–900 | 13 | 6–9 | 21–25 | 13 | 2 | 18 |
| 1b | 117–900 | 13 | 0 | 15–16 | 32 | 0 | |
| 2a | 117–665 | 18 | $2\frac{1}{2}$*–$4\frac{1}{2}$ | 23–25 | 10 | 4 | 19 |
| 2b | 117–665 | 18 | 0 | 20–21 | 29 | 13 | |

* See also Table III†. In the 6 animals with 665 µg./day there was no removal of pellets ; but at 18 months the animals were allowed to live for $2\frac{1}{2}$ months more without any new implantation of pellets. In mice the 100 per cent pellet is almost completely absorbed in about 3 months. Thus the failure of a new implantation in due time is identical with removal of pellets.

age of the animals by $2\frac{1}{2}$ to $4\frac{1}{2}$ months. Thus it would seem that it is not the age which could be responsible for the differential results with a treatment of 13 and 18 months (1b and 2b); the difference seems to be due just to the duration of treatment. Indeed, age may be responsible for a more ample growth of G induced by P; this would explain both the appearance of a macro G in 1a and the unique picture of the trifocal growth in 1a and 2a (Fig. 18 and 19). The question whether the difference between 1a and 1b is significant and whether there is an after-effect of P is certainly of fundamental pathological interest and should be studied in a much greater number of animals.

### DISCUSSION

There is no doubt that the prolonged administration of P can produce ovarian granulosa-cell tumours in mice. The question how far the incidence might depend on spontaneously occurring G in this or in other strains is of no avail because the experiments related in this paper give full evidence that the incidence of G increases (1) with the quantity of P administered, and (2) with the duration of treatment with P. In our work the administration of P was a continuous one; it is but reasonable to raise the question how far the continuity of the action of P might have contributed to the results obtained. The question is of considerable interest when discussing the tumorigenic faculties of steroids; the fibromatogenic action of oestrogens fails when the latter are administered rhythmically, i.e. discontinuously (Lipschutz, Rodríguez and Vargas, 1941; Lipschutz, 1950, pp. 40–41).

Our results give full evidence that the neoplastic faculty of P is greatly inferior to that of 19-nor-P. Even with 117 µg./day administered during 18 months the tumorigenic result is less pronounced than with 15 µg./day of 19-nor-P administered during 13 to 17 months. Thus one may argue that it was truly the " Wisdom of the Body ", to use the words of Cannon (1932), giving preference to P and not to the more active 19-nor-P; any quantitative or timing lapse would mean in the case of the latter a considerable danger for the ovary.

Granulosa-cell tumours have been produced experimentally grafting the ovary into the spleen in rats (Biskind and Biskind, 1944), in mice (Li and Gardner, 1947; Gardner, 1955, 1961; Furth and Sobel, 1947), in guinea-pigs (Mardones, Iglesias and Lipschutz, 1955; summaries Lipschutz, 1950, 1957), and in rabbits (Peckham, Greene and Jeffries, 1948; Peckham and Greene, 1952). The evolution of these experimental ovarian tumours in mice and guinea-pigs also has been studied and summarized by various authorities (Guthrie, 1957, 1959; Kullander, 1954, 1956, 1959; Lipschutz, 1957, 1960, 1963; Lipschutz, Rojas, Cerisola and Iglesias, 1960; Lipschutz, Panasevich, Cerisola and Alvarez, 1965). The final structure of G induced by 19-nor-P or by P is apparently coincident with that of G in intrasplenic grafts. However, the evolution of G originating under the influence of 19-nor-P or P is fundamentally different from that of G originating in intrasplenic ovarian grafts. In intrasplenic ovaries the growth originates in the overwhelming number of cases from cells as present in the ovarian stroma; the growth is primarily a luteoma which subsequently changes into G. On the contrary, the growth arising under the influence of the mentioned steroids is from the start a granulosa-cell tumour located, when still a microtumour, in the periphery or even on the surface of the ovary.

Since there is in some cases a proliferation of the germinal epithelium the question arises whether the latter partakes in the evolution of these steroid-induced granulosa-cell tumours. One will remember that according to Gardner (1955) even the granulosa-cell tumours in the intrasplenic grafts "appeared to arise from the germinal epithelium" (p. 116). Indeed, most of those who have worked with intrasplenic grafts were rather in favour of the cells of the stroma or of the theca being the matrix of the growth in intrasplenic ovarian grafts (Kullander, 1959; Guthrie, 1957; Lipschutz, 1960). The same seems to be true also for the microtumours originating in the kidney and the liver (Lipschutz, Panasevich and Cerisola, 1964).

The implication of P in tumorigenesis has been known for several years; evolution of mammary cancer in rats treated with a carcinogen is enhanced when P is added (Cantarow, Stasney and Paschkis, 1948; Huggins, Briziarelli and Sutton, 1959). More recently the question has been re-examined in mice by Poel (1965) with impressive results. There is no doubt that P may act as a potent co-carcinogen for mammary cancer when administered together with methylcholanthrene; incidence of the mammary tumour as induced by the carcinogen was 21 per cent but increased to 100 per cent when P was given simultaneously (Poel, 1965, p. 827). In the work of Poel, in mice no more than 2·5 $\mu$g./day of P in peanut oil were administered. In former work in rats more than 200 $\mu$g./day were injected intramuscularly (Cantarow *et al.*, 1948, p. 412), or about 25 to 30 $\mu$g./day when calculating for mice. On the other hand, our work gives full evidence that under certain quantitative and timing conditions P may produce ovarian tumours independently from any carcinogen. The tumorigenic quantities of P in absence of any carcinogen are indeed several times greater than those used in the work of the mentioned authorities.

Most interesting clinical work has been done using 17$\alpha$-hydroxyprogesterone capronate in the treatment of malignant tumours of the breast, ovary and uterus, with success in a number of cases (Jolles, 1962). At first sight this seems contradictory to the findings both of the mentioned authorities and of ours. It is of considerable interest to approach these problems from a quantitative point of view. Jolles administered to his patients 250 mg. of 17$\alpha$-hydroxyprogesterone capronate weekly by injection for up to $3\frac{1}{2}$ years. This quantity administered to patients corresponds to about 600 $\mu$g./day per 1 kg. of body weight, i.e. to about 20 $\mu$g./day in mice. A quantitative comparison with continuous absorption of P from subcutaneously implanted pellets would be difficult since the capronate of 17α-hydroxyprogesterone is more active than P. Landau, Ehrlich and Huggins (1962) administered to patients with advanced mammary cancer 50 mg./day of P intramuscularly, i.e. about 30 μg./day when calculated for mice. This quantity is not yet tumorigenic in mice (see our Table III). But here again comparison is rendered difficult because in this clinical work 5 mg. of oestradiol were given simultaneously with the mentioned 50 mg. of P. Certainly in none of these clinical studies was the treatment so prolonged as in our mice with 13 or 18 months when P becomes tumorigenic.

### SUMMARY

Granulosa-cell tumours are induced in the ovary of mice with the prolonged and continuous administration of progesterone.

The production of these tumours depends on the quantity of progesterone administered and on the duration of this administration.

The incidence increases with the amount of progesterone administered.

The neoplastic faculty of progesterone is probably ten times smaller than that of 19-nor-P.

The evolutional pattern of the steroid-induced granulosa-cell tumour is different from that of the same tumour originating in intrasplenic grafts:

(1) The steroid-induced ovarian tumour is from the start a granulosa-cell tumour whereas in grafts the same tumour is preceded by a luteoma arising from the proliferation of cells as present in the ovarian stroma.

(2) The steroid-induced granulosa-cell tumour arises in the periphery of the ovary and one cannot avoid questioning whether, besides the follicle, also the germinal epithelium partakes in this neoplastic proliferation.

Our most sincere thanks are due to Dr. Alexander Zaffaroni, of Syntex, for a generous supply of progesterone; to the technical and secretarial staff of the Institute and to the photographer Mr. Alejandro Castillo for excellent work. Our special thanks are due to the chemists of the Institute, Mr. Dušan Jadrijevic and Mrs. Silvia Girardi for tedious work on the determination of absorption from pellets in experiments of long duration.

This study was aided by a grant from the Population Council, New York.

### REFERENCES

Biskind, M. S. and Biskind, G. R.—(1944) *Proc. Soc. exp. Biol. Med.*, **22**, 176.

Cannon, W. B.—(1932) ‘ The Wisdom of the Body ’. New York (Norton & Co.).

Cantarow, A., Stasney, J. and Paschkis, K. E.—(1948) *Cancer Res.*, **8**, 412.

Fuenzalida, F.—(1950) *J. clin. Endocr. Metab.*, **10**, 1511.

Fuenzalida, F. and Lipschutz, A.—(1953) *J. clin. Endocr. Metab.*, **13**, 1201.

Furth, J. and Sobel, H.—(1947) *J. natn. Cancer Inst.*, **8**, 7.

Gardner, W. U.—(1955) *Cancer Res.*, **15**, 109. (1961) *J. natn. Cancer Inst.*, **26**, 829.

Guthrie, M. J.—(1957) *Cancer, N.Y.*, **10**, 90.—(1959) *Nature, Lond.*, **184**, 916.

Huggins, C., Briziarelli, G. and Sutton, H.—(1959) *J. exp. Med.*, **109**, 25.

Jabara, A. G.—(1959) *Aust. J. exp. Biol. med. Sci.*, **37**, 549 ; quoted from :—(1962) *Aust. J. exp. Biol. med. Sci.*, **40**, 139.

Jolles, B.—(1962) *Br. J. Cancer*, **16**, 209.

Kullander, S.—(1954) *Acta endocr., Copenh.*, Suppl. 22.—(1956) *Acta endocr., Copenh.*, Suppl. 27.—(1959) *Acta endocr., Copenh.*, **31**, 123.

Landau, R. L., Ehrlich, E. N. and Huggins, C.—(1962) *J. Am. med. Ass.*, **182**, 632.

LI, M. H. AND GARDNER, W. U.—(1947) *Science, N.Y.*, **105**, 13.—(1947) *Cancer Res.*, **7**, 549.

LIPSCHUTZ, A.—(1950) ' Steroid Hormones and Tumors '. Baltimore (Williams and Wilkins).—(1957) ' Steroid Homeostasis, Hypophysis and Tumorigenesis ' Cambridge (Heffer & Sons).—(1960) *Acta Un. int. Cancr.*, **16**, 149.—(1963) *Gynaecologia*, **156**, 93.

LIPSCHUTZ, A., IGLESIAS, R. AND SALINAS, S.—(1962) *Nature, Lond.*, **196**, 946.—(1963) *J. Reprod. Fert.*, **6**, 99.

LIPSCHUTZ, A., PANASEVICH, V. I. AND CERISOLA, H.—(1964) *Br. J. Cancer*, **18**, 655.

LIPSCHUTZ, A., PANASEVICH, V. I., CERISOLA, H. AND ALVAREZ, A.—(1964) *C.r. hebd. Séanc. Acad. Sci., Paris*, **259**, 4829.—(1965) *Revue suisse Zool.*, **72**, 99.

LIPSCHUTZ, A., RODRIGUEZ, F. AND VARGAS, L.—(1941) *Endocrinology*, **28**, 654.

LIPSCHUTZ, A., ROJAS, G., CERISOLA H. AND IGLESIAS, R.—(1960) *Acta Un. int. Cancr.*, **16**, 206.

MANDL, A. M.—(1959) *J. Endocr.*, **18**, 438, 444.

MARDONES, E., IGLESIAS, R. AND LIPSCHUTZ, A.—(1955) *Br. J. Cancer*, **9**, 409.

PECKHAM, B. M. AND GREENE, R. R.—(1952) *Cancer Res.*, **12**, 654.

PECKHAM, B. M., GREENE, R. R. AND JEFFRIES, M. E.—(1948) *Science, N.Y.*, **107**, 319.

PINCUS, G.—(1965) ' The Control of Fertility '. New York (Academic Press).

POEL, W. E.—(1965) *Br. J. Cancer*, **19**, 824.